# Apathy in schizophrenia: assessment in clinical settings and overlap with other dimensions of impairment

**DOI:** 10.1192/j.eurpsy.2021.159

**Published:** 2021-08-13

**Authors:** A. Mucci, P. Bucci, G.M. Giordano, F. Brando, S. Galderisi

**Affiliations:** Department Of Psychiatry, Univeristy of Campania Luigi Vanvitelli, Naples, Italy

**Keywords:** Avolition, negative symptoms, apathy, Primary negative symptoms

## Abstract

Negative symptoms are considered a core feature of schizophrenia. They are present since the prodromal phase and tend to persist more than other psychopathological dimensions in the chronic stages. The domain of apathy has attracted research efforts for the strong association with poor functional outcome. This negative symptom domain is observed in a number of neuropsychiatric disorders and might have both overlapping and distinct pathophysiological mechanisms. In schizophrenia it can be secondary to other aspects of the disorder, such as positive symptoms and depression, to drug side effects and/or social isolation, often observed in affected subjects. When primary to schizophrenia, apathy is conceptualized in terms of a reduction of the voluntary activity due to a lack of interest and motivation for goal-directed behavior initiation and persistence. In a percentage of subjects, apathy tend to persist and do not respond to available pharmacological and psychosocial treatments. The assessment of this domain in patients with schizophrenia using internationally recognized criteria for its definition, as were recently developed in other neuropsychiatric disorders, might help disentangle the different pathophysiological mechanisms. In the presentation, studies of apathy in schizophrenia will be illustrated to highlight the relationships with cognitive dysfunction, other psychopathological dimensions and functional outcome using state of the art instruments to assess the construct in schizophrenia.

**Disclosure:**

Prof. Mucci has been a consultant and/or advisor to or has received honoraria from Gedeon Richter Bulgaria, Janssen Pharmaceuticals, Lundbeck, Otsuka, Pfizer and Pierre Fabre.

